# Machine Learning Modeling of Hospital Length of Stay After Breast Cancer Surgery: Comparison of Random Forest and Linear Regression Approaches

**DOI:** 10.3390/medicina62010088

**Published:** 2025-12-31

**Authors:** Iulian Slavu, Raluca Tulin, Alexandru Dogaru, Ileana Dima, Cristina Orlov Slavu, Daniela-Elena Gheoca Mutu, Adrian Tulin

**Affiliations:** 1 Faculty of Medicine, University of Medicine and Pharmacy Carol Davila, 050474 Bucharest, Romania; 2 General Surgery Department, Agrippa Ionescu Emergency Clinical Hospital, 011356 Bucharest, Romania; 3 Endocrine Department, Agrippa Ionescu Emergency Clinical Hospital, 011356 Bucharest, Romania; 4 Oncology Department, Agrippa Ionescu Emergency Clinical Hospital, 011356 Bucharest, Romania; 5 Plastic Surgery Department, Agrippa Ionescu Emergency Clinical Hospital, 011356 Bucharest, Romania

**Keywords:** breast cancer, breast surgery, hospital stay, machine learning, predictive modeling, postoperative outcomes, surgical oncology, enhanced recovery, clinical prediction models, real-world data

## Abstract

*Background and Objectives*: Hospital length of stay (LOS) after breast cancer surgery is a key indicator of postoperative recovery, healthcare quality, and hospital resource utilization. Traditional statistical approaches have identified general correlates of LOS but remain limited in predictive accuracy, particularly in heterogeneous real-world surgical populations. Machine learning (ML) models may offer improved performance by capturing nonlinear interactions among clinical, pathological, and operative factors. This study aimed to evaluate ML algorithms for LOS prediction and to identify determinants of prolonged hospitalization in a contemporary breast cancer cohort. *Materials and Methods*: We conducted a retrospective cross-sectional study of 198 consecutive breast cancer patients who underwent surgery between January 2022 and December 2023 at a single tertiary care center. Clinical, pathological, and surgical data were extracted from electronic medical records. Three regression models—multiple linear regression, Random Forest, and Gradient Boosting—were trained to predict continuous LOS, and three classification models were applied to prolonged LOS (≥10 days). Model performance was assessed using mean absolute error (MAE), root mean square error (RMSE), coefficient of determination (R^2^), and area under the curve (AUC). Feature importance was analyzed for the best-performing model. *Results*: The median LOS was 7 days (IQR 5–10), ranging from 1 to 26 days. Breast-conserving surgery showed the shortest LOS (median 3 days), while mastectomy with immediate reconstruction resulted in the longest stays (median 8 days). Random Forest regression achieved the lowest prediction error (MAE 2.31 days; RMSE 2.82; R^2^ = 0.37), outperforming Gradient Boosting and substantially surpassing linear regression (MAE 8.63 days; R^2^ = –8.17). Key predictors included age, surgical complexity, reconstruction modality, BMI, implant capacity, and tumor burden. Classification models yielded modest AUCs (0.545–0.589) with low sensitivity, indicating limited discriminative performance for dichotomized LOS outcomes. *Conclusions*: Machine-learning models, particularly Random Forest, substantially improve LOS prediction compared with classical regression and provide clinically meaningful insights into the drivers of hospitalization after breast cancer surgery. Continuous LOS modeling is more informative than binary thresholds. These findings support integrating ML-based tools into perioperative planning, resource allocation, and patient counseling in breast surgical care.

## 1. Introduction

Hospital length of stay (LOS) following breast cancer surgery represents a critical metric for evaluating healthcare efficiency, perioperative quality, patient recovery, and resource utilization. As treatment pathways diversify—with increasing use of oncoplastic procedures, immediate reconstruction, and selective axillary surgery—the variability in LOS has grown considerably. Beyond its clinical significance, LOS is also a major driver of hospital expenditures. In many European health systems, each additional postoperative day incurs substantial direct and indirect costs, consuming bed capacity and staff resources while limiting access for other patients. Even modest reductions in LOS have been associated with meaningful decreases in surgical oncology spending and improved operational efficiency, making accurate prediction highly relevant for both clinicians and administrators.

Several studies have examined determinants of hospital length of stay (LOS) after breast surgery, though most have relied on classical statistical approaches with limited predictive accuracy. For example, Kotha et al., using a national NSQIP cohort of 9686 autologous breast reconstructions, showed that factors such as reconstruction type, BMI, diabetes, and operative time were significantly associated with extended LOS, but their multivariable logistic regression model explained only part of the observed variability [1].

Machine learning (ML) offers a promising alternative. ML algorithms can capture nonlinear and interactive effects across clinical, pathological, and operative variables that traditional statistical models often miss. In oncologic surgery, ML methods have repeatedly outperformed classical tools in predicting postoperative outcomes. For example, Rubenstein et al. (2023) applied ML to national breast reconstruction data and demonstrated superior performance of gradient-boosting models over logistic regression for predicting early postoperative complications and resource use [2].

More broadly, Xue et al. found that ML models achieved substantially higher AUROCs than logistic regression for predicting major postoperative complications, demonstrating the scalability and accuracy of ML-based perioperative risk prediction [3].

Similarly, Explainable predictions of a machine learning model to forecast the postoperative length of stay for severe patients was used on a set of approximately 240,000 surgical patients and demonstrated that ML models (e.g., XGBoost) can predict postoperative length of stay (LOS) with a mean error of around 3 days, highlighting the practical potential of ML for discharge planning [4,5].

Finally, a recent narrative review on reconstruction surgery, Artificial Intelligence in Breast Reconstruction: Enhancing Surgical Planning, Outcome Prediction, and Patient Engagement, describes how ML can predict postoperative complications and patient-reported outcomes in breast reconstruction, although it notes that no published studies to date have combined tumor biology, axillary status, reconstruction details, and neoadjuvant therapy into a unified predictive framework for LOS specifically [6].

Previous machine-learning studies of postoperative length of stay in breast surgery have largely focused on reconstruction-only cohorts or registry-based datasets with limited oncologic and biological detail. In contrast, the present study applies machine-learning models to a real-world breast cancer cohort encompassing the full spectrum of surgical procedures and integrates oncologic, axillary, reconstructive, and biological variables within a single predictive framework. This approach enables a more comprehensive representation of clinical complexity than prior analyses.

Given these gaps, we aimed to evaluate whether machine learning could enhance prediction of hospital LOS after breast surgery in a real-world cohort from our institution. Our center performs a broad spectrum of breast operations—from conservative lumpectomies to complex mastectomies with immediate reconstruction—providing an ideal setting to assess LOS across diverse surgical pathways. From a practical perspective, improved LOS prediction has direct implications for cost containment, bed management, staffing optimization, and patient counseling. From an academic perspective, this area remains underexplored and requires modern analytical approaches capable of handling clinical complexity. This integrated modeling strategy represents a clinically novel application of machine learning for length-of-stay prediction in breast cancer surgery.

Thus, in this study, we developed and internally validated ML models to predict LOS using clinical, pathological, and surgical variables collected from breast cancer patients operated between 2022 and 2023. By comparing ML performance with classical regression and identifying the strongest predictors of LOS, we sought to generate clinically actionable insights and contribute novel methodological perspectives to the existing breast surgery literature.

## 2. Materials and Methods

We performed a retrospective cross-sectional study to evaluate the ability of machine learning models to predict postoperative hospital length of stay (LOS) after breast cancer surgery, and to identify the clinical, pathological, and procedural determinants influencing hospitalization duration. The manuscript was prepared in accordance with STROBE guidelines for observational studies. The study population consisted of consecutive patients (n = 198) undergoing breast surgery in our department between January 2022 and December 2023.

The study was approved by the “Prof. Dr. Agrippa Ionescu” Clinical Emergency Hospital Ethics Committee (Approval No. 112/20 February 2023). All patients included in the analysis had previously signed an institutional informed consent for the use of clinical data for research purposes. The study adhered to the principles of the Declaration of Helsinki.

Inclusion criteria: Adult patients (≥18 years), Pathologically confirmed breast cancer, Surgical treatment including breast-conserving surgery, mastectomy, or mastectomy with immediate reconstruction, Availability of key clinical, pathological, and surgical variables, Complete hospitalization data (dates of admission, discharge, and operative procedures).

Exclusion criteria: Benign or noninvasive lesions (e.g., pure DCIS without invasive component), Palliative or incomplete oncologic surgery, Missing LOS data or incomplete surgical records, Missing essential predictors (e.g., absent pathology report, unknown surgical procedure), Prophylactic mastectomy without concurrent malignancy.

Data Source and Variables

Data were extracted from the electronic medical records of breast cancer patients treated in a multidisciplinary setting at “Prof. Dr. Agrippa Ionescu” Clinical Emergency Hospital, Bucharest, Romania. All information was derived from the institutional database containing demographic, clinical, pathological, and surgical variables, as well as hospitalization timelines.

Missing continuous variables were imputed using the median and categorical variables using the most frequent category to ensure a complete feature matrix compatible with machine-learning algorithms.

Statistical Analysis

Throughout this manuscript, the term ‘machine learning’ is used to describe the specific predictive algorithms applied, while ‘artificial intelligence’ is used only in a broader conceptual context.

All statistical analyses were carried out using Python (version 3.10) in a Jupyter Notebook environment. Data manipulation, cleaning, and preprocessing were performed with the pandas and numpy libraries. Machine-learning methods and classical regression techniques were implemented using the scikit-learn library. Before analysis, all continuous variables—including age, BMI, Ki-67 index, and tumor size—were converted to numeric format and screened for outliers or implausible entries. Missing values in continuous variables were imputed using the median, whereas categorical missing values were replaced with the most frequent category. All categorical predictors were encoded through one-hot encoding. Administrative data, patient identifiers, and date fields were excluded from feature sets used for modeling.

Continuous variables were summarized using median and interquartile range (IQR), while categorical variables were presented as counts and percentages. Model performance metrics were compared descriptively. No formal hypothesis testing was performed for model comparisons, as the primary aim was predictive performance rather than inferential significance.

Missing continuous variables were imputed using the median and categorical variables using the most frequent category. Missingness indicators were not included as separate features, as missing values were considered primarily reflective of retrospective documentation practices rather than informative clinical absence, and to limit feature-space expansion in a moderate-sized dataset.

The category ‘other surgical combinations’ was used to group heterogeneous procedures that could not be reliably classified as breast-conserving surgery, mastectomy alone, or mastectomy with immediate reconstruction.

The primary outcome of interest was hospital length of stay (LOS), defined as the total number of postoperative days spent in the hospital. LOS was summarized using medians and interquartile ranges, and differences between surgical groups were assessed using nonparametric tests such as the Mann–Whitney U test. When more than two groups were compared, the Kruskal–Wallis test was applied. Correlations between LOS and continuous predictors were examined using Spearman’s rank correlation coefficient.

To predict LOS as a continuous outcome, three models were developed: multiple linear regression, a Random Forest regressor, and a Gradient Boosting regressor. The dataset was randomly split into an 80% training set and a 20% test set. Model fitting and parameter tuning were performed exclusively within the training set using repeated five-fold cross-validation. Performance was assessed on the held-out test set using mean absolute error (MAE), root mean square error (RMSE), and the coefficient of determination (R^2^). Cross-validated MAE was also computed to evaluate internal consistency across folds. For interpretation, feature importance values were extracted from the Random Forest model to identify the variables contributing most substantially to LOS predictions.

A secondary analysis examined prolonged LOS, defined a priori as ten days or more. A binary variable was created to reflect this threshold, and three classification models—logistic regression, Random Forest classifier, and Gradient Boosting classifier—were trained using the same 80/20 split.

Prolonged LOS was defined a priori as hospitalization of ten days or more, corresponding to the upper quartile of LOS distribution in the study cohort and reflecting institutionally prolonged postoperative recovery.

Model discrimination was evaluated using the area under the receiver operating characteristic curve (AUC), overall accuracy, sensitivity, specificity, and confusion matrices. Cross-validated AUC was computed using five-fold cross-validation within the training dataset. Predicted probabilities of 0.5 or greater were used as the default classification threshold for binary predictions.

Model performance metrics were derived from internal validation only, using a held-out test set and five-fold cross-validation within the training data. Cross-validated results were consistent across folds, indicating stable model performance.

Although the sample size was moderate (n = 198), non-linear ensemble models were selected because postoperative LOS is influenced by complex interactions among demographic, oncologic, and surgical variables that violate linear assumptions. To mitigate overfitting, model complexity was controlled through repeated cross-validation, evaluation on a held-out test set, and comparison with a linear regression baseline. Random Forest was favored due to its robustness in moderate-sized clinical datasets and its ability to model nonlinear effects without extensive hyperparameter tuning.

A total of 23 predictors were included prior to preprocessing, encompassing demographic, clinical, tumor-related, genetic, and surgical variables; categorical predictors were subsequently transformed using one-hot encoding, increasing the effective feature dimensionality. Categorical variables were transformed using one-hot encoding, increasing the effective feature dimensionality. To limit overfitting in this moderate-sized cohort, model training incorporated repeated five-fold cross-validation within the training set and performance evaluation on a held-out test set.

All preprocessing steps, modeling functions, and validation procedures were implemented using Python 3.10 with scikit-learn (version 1.2 or higher), pandas (version 1.5 or higher), and numpy (version 1.23 or higher). Full reproducibility of the analysis is ensured through the availability of the original Python code upon request.

## 3. Results

### 3.1. Study Population

A total of 198 patients with complete data on hospital length of stay (LOS) were included in the analysis. The median age was 53.5 years (IQR 45.0–65.0), with an age range of 29–87 years. The median BMI was 26.1 kg/m^2^ (IQR 22.8–29.8) (Table 1).

Cardiovascular comorbidities were present in approximately 49.0% of patients, and 23.7% had diabetes mellitus. Most tumors were hormone receptor–positive: among patients with available immunohistochemistry results, 84.8% were ER-positive and 81.2% were PR-positive, while 20.2% were HER2-positive. The Ki-67 proliferation index was available for 118 patients, with a median Ki-67 of 20% (IQR 10–30%); 51.7% of those with available data had high Ki-67 ≥ 20% (Table 1).

Neoadjuvant chemotherapy was administered in 85 patients (42.9%), while 102 patients (51.5%) did not receive neoadjuvant chemotherapy. For the remaining cases, neoadjuvant status was uncertain or recorded as radiotherapy only (Table 1).

Other surgical combinations include heterogeneous or combined procedures not fitting predefined surgical categories, including bilateral procedures, combined oncologic–reconstructive operations not meeting the predefined criteria for immediate reconstruction, or atypical/staged surgical sequences.

### 3.2. Surgical Treatment and Axillary Management

Breast-conserving surgery (BCS) was performed in 18 patients (9.1%). Mastectomy without immediate reconstruction was recorded in 60 patients (30.3%), while 40 patients (20.2%) underwent mastectomy with immediate reconstruction according to our operational definition (principal procedure including “MMM” and a documented immediate reconstructive procedure). The remaining patients had other combinations or complex procedures that did not clearly fall into these three major categories.

Sentinel lymph node biopsy (SLNB) using a documented technique (“DA” in the sentinel node field) was performed in 51 patients (25.8%), whereas the field indicated “NU” (no SLNB) in 87 patients (43.9%); the remainder had “R” or “P” codes suggesting alternative or repeated procedures. Axillary lymphadenectomy was performed in 90 patients (45.5%) (“DA” or “DA—in alt timp operator”), while 47 patients (23.7%) had a clearly documented absence of axillary dissection (“NU”).

### 3.3. Distribution of Hospital Length of Stay

The median LOS for the entire cohort was 7 days (IQR 5–10), with a range of 1 to 26 days. The distribution of LOS was right-skewed, with a concentration around 5–7 days.

Marked differences in LOS were observed between surgical groups:

- Breast-conserving surgery (BCS):
  median LOS 3 days (IQR 2–3), n = 18
- Mastectomy without immediate reconstruction:
  median LOS 7 days (IQR 5–10), n = 60
- Mastectomy with immediate reconstruction:
  median LOS 8 days (IQR 6.8–11.3), n = 40

BCS was associated with significantly shorter LOS than mastectomy without reconstruction (*p* < 0.001, Mann–Whitney U test). Among mastectomy patients, those with immediate reconstruction had a significantly longer LOS compared to those without reconstruction (*p* ≈ 0.03).

Axillary lymphadenectomy was also associated with numerically longer hospitalization. Patients who underwent lymphadenectomy had a median LOS of 7 days (IQR 5–10; n = 90), compared with 6 days (IQR 3–9; n = 47) for those without lymphadenectomy, with a statistical trend (*p* ≈ 0.07). Neoadjuvant chemotherapy did not significantly affect LOS: patients with and without neoadjuvant treatment both had a median LOS of 7 days (*p* ≈ 0.18).

### 3.4. Regression Models for LOS Prediction (Primary Outcome)

Three models were developed to predict LOS as a continuous variable: multiple linear regression, Random Forest regression, and Gradient Boosting regression. All models used an identical set of clinical, pathological, and surgical predictors and were constructed with standardized preprocessing steps that included imputation of missing values and one-hot encoding of categorical variables. When applied to the test set representing 20% of the dataset, clear performance differences emerged among the three approaches. Linear regression performed poorly, yielding a mean absolute error (MAE) of 8.63 days, a root mean square error (RMSE) of 10.73 days, and a markedly negative coefficient of determination (R^2^ = –8.17), indicating substantial model misfit. Its cross-validated MAE was 5.46 days, further reinforcing the inadequacy of a purely linear method in this heterogeneous clinical dataset.

By contrast, the Random Forest regressor demonstrated the strongest predictive capacity, with an MAE of 2.31 days, an RMSE of 2.82 days, and an R^2^ of 0.37 on the test set. Its cross-validated MAE was 3.04 days, confirming consistent performance across multiple folds. The Gradient Boosting model also performed reasonably well, with an MAE of 2.63 days, RMSE of 3.15 days, and R^2^ of 0.21, and a cross-validated MAE of 3.07 days. However, although Gradient Boosting approached the Random Forest in error magnitude, its explained variance was lower. Overall, the Random Forest model achieved the lowest prediction error and the highest degree of explained variability, with an average absolute error of approximately two to three days. In contrast, the poor performance of the linear regression model, reflected in both high error metrics and a strongly negative R^2^, highlights the limitations of linear assumptions when modeling LOS in a real-world, clinically diverse patient population (Table 2).

This scatter plot illustrates the relationship between predicted and actual postoperative length of stay (LOS) generated by the Random Forest regression model. Each point represents an individual patient from the test dataset. The diagonal identity line (y = x) denotes perfect agreement between predicted and observed LOS values.

**Figure 1:**
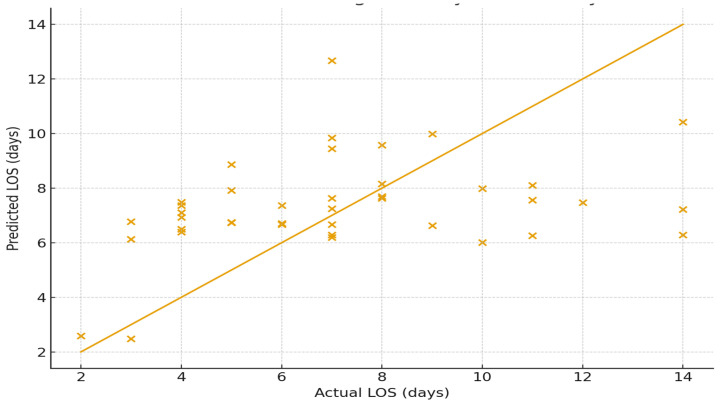
Predicted vs. Actual Length of Stay with Identity Line.

Points lying close to this line indicate accurate predictions, whereas deviations reflect model error (Figure 1). The clustering of observations around the identity line, particularly within the 5–10-day range—which represents the most common LOS interval in the cohort—demonstrates that the Random Forest model provides reasonably strong predictive performance for typical cases. Greater dispersion is visible at higher LOS values, indicating increasing prediction difficulty for more complex or prolonged hospitalizations, a trend consistent with the model’s R^2^ value. Overall, the figure highlights the capacity of machine learning to approximate real-world LOS with moderate accuracy while emphasizing the inherent variability associated with predicting extended postoperative recovery.

### 3.5. Feature Importance in the Best Regression Model

Feature importance analysis from the best-performing regression model, the Random Forest regressor, demonstrated that both patient-related and surgery-related variables substantially contributed to LOS prediction. Age emerged as the single most influential predictor, suggesting a strong relationship between patient age and recovery trajectory after surgery. Several surgical variables were also highly impactful, particularly those reflecting the type and complexity of the primary procedure. These included distinctions between breast-conserving surgery and mastectomy, the presence of bilateral submuscular implants, and combined reconstructive approaches incorporating both implants and latissimus dorsi flaps.

Body habitus parameters, including BMI and body weight, contributed meaningfully to model performance, as did BRCA status, where “BRCA” (no genetic testing result recorded) had a notable effect relative to known mutation status. Among reconstruction-specific attributes, implant capacity (volume) in immediate reconstructions played a substantial predictive role. Tumor-related variables, including the Ki-67 proliferation index and pathological tumor size (pT), were also influential. In contrast, classical tumor biology markers—such as ER, PR, and HER2 receptor status—displayed relatively low importance in determining LOS. Together, these results suggest that operative complexity, patient constitution, and tumor burden exert greater influence on LOS than receptor profile, with age and surgical parameters dominating the predictive landscape (Table 3).

**Figure 2:**
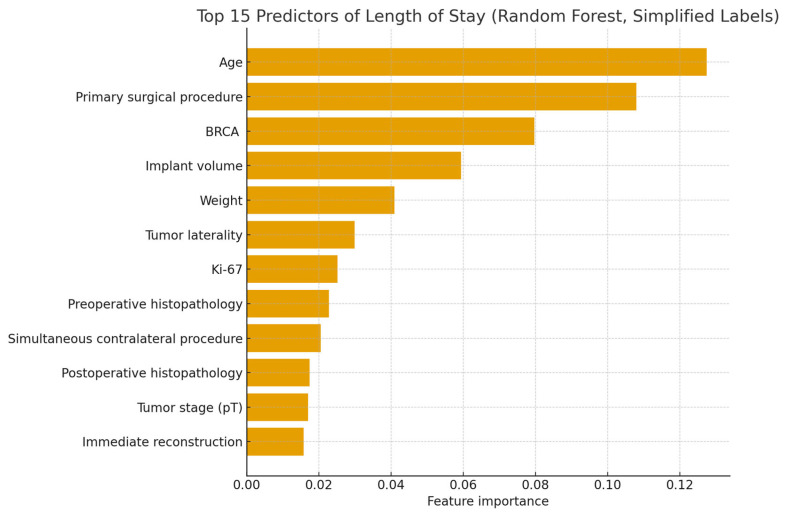
Top 15 Predictors of Length of Stay (Random Forest, Simplified Labels).

Age emerged as the strongest individual predictor, followed by the primary surgical procedure, BRCA status, implant volume, and patient weight. Several clinically relevant tumor-related and procedural variables—including tumor laterality, Ki-67 proliferation index, preoperative and postoperative histopathology, simultaneous contralateral surgery, tumor stage (pT), and immediate reconstruction—also contributed meaningfully, although with lower relative importance. The ranking highlights that LOS is determined by a combination of patient-level factors, operative complexity, and tumor burden, rather than by histopathological markers alone. These findings support the model’s emphasis on surgical and physiological attributes as the primary drivers of postoperative recovery duration (Figure 2).

This figure displays the relative importance of the top 15 predictors contributing to postoperative length of stay (LOS) in the Random Forest regression model. Higher bars indicate variables with greater influence on LOS prediction.

The Random Forest model achieved moderate predictive performance (R^2^ = 0.37), indicating partial but clinically meaningful explanation of LOS variability rather than overfitting or overly optimistic accuracy

### 3.6. Classification Models for Prolonged LOS (Secondary Outcome)

Prolonged LOS was defined a priori as a hospitalization of ten days or more, a threshold met by 58 patients (29.3% of the cohort). Three classification models were constructed to predict prolonged stay: logistic regression, Random Forest classification, and Gradient Boosting classification. Their test-set performance showed modest discrimination overall. Logistic regression achieved an AUC of 0.545, an accuracy of 0.65, a sensitivity of 0.00, and a specificity of 0.93, with a cross-validated AUC of 0.535. This model favored specificity but failed to correctly identify patients with prolonged LOS.

The Random Forest classifier slightly improved discrimination, achieving an AUC of 0.589 and an accuracy of 0.675, but again demonstrated a sensitivity of 0.00, identifying none of the prolonged-stay cases at the conventional probability threshold. Specificity was high at 0.96, and cross-validated AUC reached 0.660. The Gradient Boosting classifier performed similarly, with an AUC of 0.579, accuracy of 0.675, sensitivity of 0.17, and specificity of 0.89, along with a cross-validated AUC of 0.654. While Gradient Boosting showed slightly improved sensitivity compared with the other models, overall discriminatory ability remained limited. Taken together, these findings indicate that predicting dichotomized LOS is considerably more difficult than modeling LOS as a continuous variable. The consistently low sensitivity across models suggests that the binary definition of prolonged stay captures complex multivariate interactions that are not easily separable using the available predictors, whereas continuous LOS modeling retains a richer representation of postoperative variability (Table 4).

**Figure 3:**
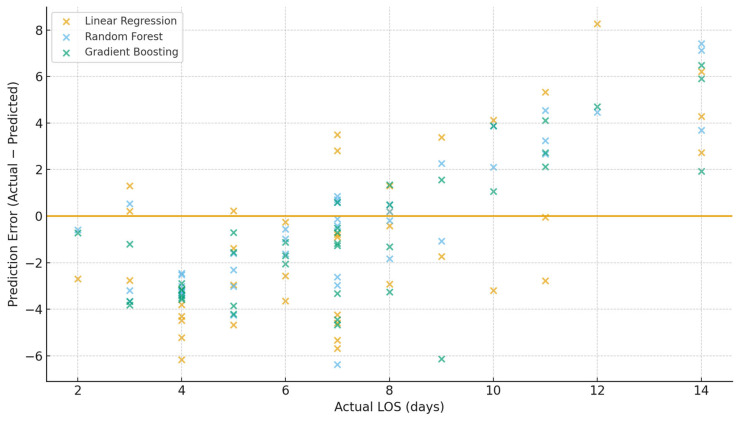
Classification Model Performance for Prolonged LOS (≥10 days).

**Figure 4:**
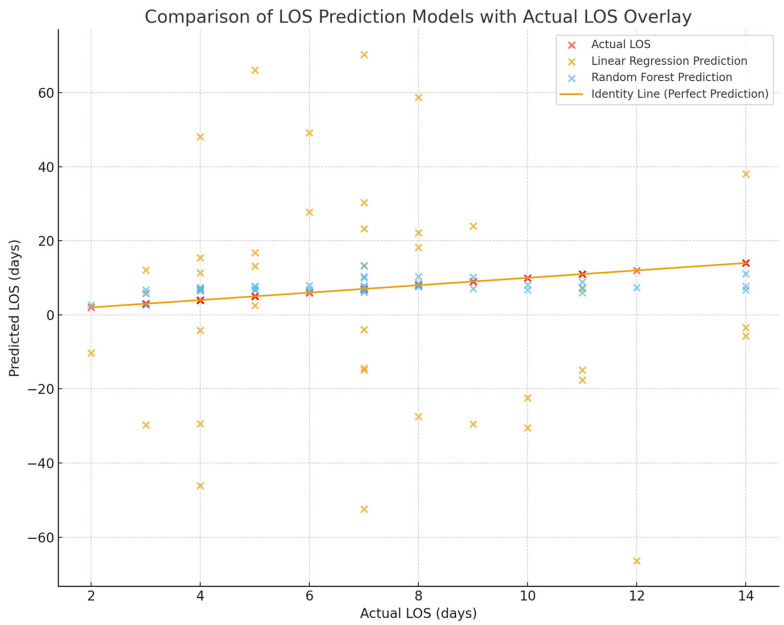
Comparison of LOS Prediction Models with Actual LOS Overlay.

The clustering of Random Forest predictions close to the identity line indicates superior predictive accuracy and calibration, with errors remaining small and symmetrically distributed around the true LOS values. In contrast, the linear regression predictions exhibit broad dispersion and systematic deviations from the identity line, reflecting substantial underfitting and poor modeling of nonlinear relationships within the heterogeneous clinical data. This visualization clearly demonstrates that Random Forest regression markedly outperforms linear regression in estimating postoperative LOS and provides more reliable predictions for real-world clinical use (Figure 3 and Figure 4).

The above figure shows prediction errors (actual minus predicted length of stay) for three regression models—multiple linear regression, Random Forest regressor, and Gradient Boosting regressor. Each point represents a single patient in the test dataset. The horizontal line at zero denotes perfect prediction. Random Forest demonstrates the smallest dispersion of errors, consistent with its superior MAE, RMSE, and R^2^ values.

This scatter plot compares the performance of multiple linear regression and Random Forest regression models in predicting postoperative length of stay (LOS) following breast cancer surgery. Red points represent the actual LOS values derived from the institutional dataset and illustrate the real distribution of hospitalization duration in the cohort. Blue points correspond to predictions generated by the Random Forest model, while yellow points represent predictions from the multiple linear regression model. The diagonal identity line denotes perfect agreement between predicted and observed LOS.

### 3.7. Summary of Key Findings

In this real-world cohort of 198 patients undergoing breast surgery, LOS was shortest among those who underwent breast-conserving surgery, with a median of 3 days. Patients receiving mastectomy without immediate reconstruction had a longer median stay of 7 days, while those undergoing mastectomy with immediate reconstruction experienced the longest stays, with a median of 8 days. Machine-learning models demonstrated notable advantages over classical linear regression in predicting LOS. Specifically, the Random Forest regressor achieved a mean absolute error of 2–3 days and outperformed both linear and Gradient Boosting approaches, reflecting its ability to capture nonlinear relationships and interactions among the diverse clinical and surgical variables present in this dataset. Feature importance patterns emphasized the substantial influence of age, surgical complexity, body habitus, and tumor burden—particularly pathological tumor size and Ki-67—whereas receptor status contributed relatively little. Classification models designed to predict prolonged LOS of ten days or more demonstrated only modest discriminative ability and consistently low sensitivity, underscoring the challenges of using binary thresholds to represent inherently continuous recovery patterns. Overall, the findings indicate that continuous LOS prediction provides more accurate, stable, and clinically meaningful results than dichotomized modeling, and they confirm the relevance of machine-learning approaches in understanding and forecasting postoperative trajectories in breast surgery patients.

## 4. Discussion

In this retrospective cross-sectional study, we evaluated postoperative hospital length of stay (LOS) after breast cancer surgery using real-world data and modern machine-learning (ML) techniques. The primary aim was to determine whether ML could more accurately predict LOS than traditional statistical approaches, and the secondary aim was to identify which clinical and surgical factors most strongly influence hospitalization duration. Prior work has shown that ML can indeed improve LOS prediction in surgical and oncologic settings. For example, Jo YY et al. in their study entitled “Prediction of Prolonged Length of Hospital Stay After Cancer Surgery Using Machine Learning on Electronic Health Records” used EHR from 42,751 cancer-surgery patients (multiple cancer types, including breast) and found that ML methods (e.g., gradient boosting) modestly outperformed logistic regression in predicting prolonged LOS [6].

Similarly, Cho et al. in their study named “Explainable predictions of a machine learning model to forecast the postoperative length of stay for severe patients” developed ML regression models (including Random Forest and XGBoost) on a cohort of ~240,000 patients and demonstrated that ML can predict postoperative LOS with a mean error of around 3 days—highlighting the potential of non-linear models in LOS forecasting [4].

Moreover, in a more specific breast-surgery context Gabay et al. in their study “Predicting Breast Reconstruction Readmission, Reoperation, and Prolonged Length of Stay: A Machine Learning Approach” showed that ML models applied to breast reconstruction patients (implant, autologous, and tissue-expander cohorts) could predict “prolonged LOS” with AUC up to ~0.86 depending on reconstruction type [7].

Finally, a recent single-center study by Trufio et al., “Flow Analysis of Mastectomy Patients Using Length of Stay”, implemented both regression and classification models on 1123 mastectomy patients and demonstrated that non-linear models (polynomial regression) can effectively characterize LOS using clinical and organizational variables [8].

### 4.1. Interpretation of Primary Endpoint: Predicting LOS Using Machine Learning

Across all three predictive algorithms tested, Random Forest demonstrated the best performance, achieving a mean absolute error of 2.31 days, an RMSE of 2.82, and an R^2^ of 0.37. In contrast, multiple linear regression—despite its widespread use in medical literature—performed poorly, with an MAE of 8.63 days and a markedly negative R^2^ (–8.17). These results highlight the inherent limitations of linear modeling when applied to heterogeneous real-world surgical data, where complex nonlinear relationships are the rule rather than the exception. Our findings are consistent with those of Kotha et al., who used the ACS-NSQIP database to analyze 9686 free-flap breast reconstructions and showed that, although multivariable regression identified several risk factors for extended LOS (e.g., higher BMI, diabetes, malignancy history, prolonged operative time, and immediate postmastectomy reconstruction), substantial variability in LOS persisted despite adjustment, underscoring the difficulty of capturing complex interactions with traditional linear models [1].

The poor performance of multiple linear regression is best interpreted as a limitation of linear assumptions in modeling postoperative LOS rather than suboptimal model specification, as identical predictors and preprocessing steps were applied across all models. Capturing such relationships with linear regression would require extensive manual feature engineering and interaction modeling, which is neither scalable nor robust in moderate-sized clinical datasets.

Likewise, Rubenstein et al., in a national analysis of 2019–2020 breast reconstruction cases, reported that standard regression-based models linked shorter LOS to temporal practice changes and reconstructive technique, yet notable unexplained variation remained across patient subgroups and institutions, highlighting the need for more flexible modeling frameworks—such as machine learning—for robust LOS prediction [2].

Random Forest’s strong performance is attributable to its ability to integrate high-dimensional clinical data, model nonlinear effects, and handle interactions between variables without prespecified assumptions. As seen in our feature-importance analysis, age, surgical complexity, implant-related variables, BRCA status, BMI, and tumor size were among the dominant predictors, whereas classical tumor biology markers contributed less substantially.

Given the sample size (n = 198), overfitting remains a potential concern, particularly for tree-based models after one-hot encoding. However, consistent cross-validated performance and moderate explained variance suggest that the models captured clinically meaningful patterns rather than noise. All results reflect internal validation only and should be interpreted as exploratory.

As a single-center study, postoperative length of stay is influenced by institution-specific factors, including discharge policies, perioperative care pathways, reconstruction availability, and local healthcare system constraints. These organizational characteristics may differ substantially across centers and countries, potentially limiting direct generalizability of LOS estimates and model performance. Accordingly, the reported LOS values and predictive performance should be interpreted as context-dependent rather than universally applicable.

### 4.2. Interpretation of Secondary Endpoint: Prolonged LOS and Classification Model Performance

In the secondary analysis, classification models showed modest discrimination for predicting prolonged LOS (≥10 days): Random Forest achieved an AUC of 0.589, while Gradient Boosting reached 0.579, both substantially higher than logistic regression (AUC 0.545). However, all models suffered from low sensitivity, indicating difficulty in identifying patients at risk for extended hospitalization. This suggests that prolonged LOS may be a challenging binary outcome to predict reliably—potentially because hospital stay reflects a continuous recovery spectrum, and dichotomizing it (e.g., “≥10 days”) may mask important clinical variability.

The low sensitivity observed in classification models underscores the difficulty of predicting dichotomized LOS outcomes and highlights the limitations of applying arbitrary thresholds to a continuous recovery process. These findings support the use of continuous LOS modeling as a more informative and clinically relevant approach.

The superior performance of Random Forest compared with linear regression—despite the moderate sample size—underscores the nonlinear nature of LOS determinants in breast surgery. The poor performance of linear regression further supports the need for flexible modeling approaches, even in smaller real-world datasets, when appropriate validation strategies are employed.

### 4.3. Comparison with Existing Literature

Only a limited number of studies have applied machine-learning (ML) or other advanced modeling approaches to postoperative outcomes in breast surgery. For example, Kotha et al. (2021) analyzed 9686 free-flap breast reconstruction cases in a national cohort and found that conventional multivariable regression identified predictors of extended hospital stay but still left substantial unexplained variability—underscoring limitations of linear models in complex surgical populations [1].

Similarly, Rubenstein et al. (2023) examined national data from breast reconstruction patients and reported wide variability in length of stay that persisted after standard regression adjustment, highlighting the need for more flexible modeling methods capable of capturing nonlinear interactions [2].

Our analysis adds novel evidence by demonstrating ML’s superiority specifically for predicting LOS after breast cancer surgery, using a detailed real-world dataset that integrates biological, anatomical, treatment-based, and surgical variables—an approach that has not been previously reported in the breast-surgery literature.

### 4.4. Why LOS Matters: Clinical, Economic, and Operational Impact

Length of stay (LOS) is both a clinical marker of postoperative recovery and a major determinant of hospital resource utilization. Published health-economic analyses show that hospital-day costs vary widely across countries and levels of care. Recent OECD data estimate that an acute-care inpatient day in Western Europe typically costs €400–€650 depending on staffing intensity and case complexity (OECD Health Statistics 2023—Average cost per hospital day) [9].

Meanwhile, Eastern European systems report substantially lower—but still meaningful—costs: for example, Romanian National Health Insurance (CNAS) DRG reimbursement tables imply an average €100–€180 per inpatient day when dividing total DRG tariff by median LOS for general surgical admissions (CNAS DRG Tariffs, 2023—Casa Națională de Asigurări de Sănătate) [10].

In the United States, hospital inpatient days are considerably more expensive. A large national analysis estimated the average cost of a medical–surgical hospital day at $2300–$2900, with oncology and postsurgical units at the upper end due to monitoring and nurse-to-patient ratios (Agency for Healthcare Research and Quality, HCUP Statistical Brief 268, 2021) [11].

In this context, even a 1–2 day reduction in LOS per patient translates into meaningful annual savings, improved bed turnover, and increased capacity for urgent or oncologic admissions. At our own institution, administrative reports indicated that breast-surgery admissions generated unexpectedly high total expenditures—at times exceeding those for more complex gastrointestinal procedures—which motivated this investigation into the determinants of prolonged hospitalization.

### 4.5. Clinical Interpretation of Our Findings

Our results show that patients undergoing mastectomy with immediate reconstruction experience the longest LOS (median 8 days), in line with large series of autologous and complex breast reconstruction where hospital stays of 3–7 days are common and extended LOS is driven by patient and operative risk factors. For example, Kotha et al. used a national NSQIP cohort of 9686 free-flap reconstructions and found that immediate post-mastectomy reconstruction, higher BMI, diabetes, and longer operative time were all independently associated with extended LOS (>5 days) [1].

Similarly, fast-track or ERAS-style programs for autologous reconstruction report baseline LOS around 6–7 days, with structured perioperative pathways reducing this by about one day or more [12].

In contrast, breast-conserving surgery is typically performed as outpatient or 23 h short-stay surgery, with very short hospital stays: a systematic review of day surgery for breast cancer showed high same-day discharge rates and low readmission while randomized and observational studies of breast-conserving surgery with sentinel node biopsy confirm the feasibility and safety of day surgery compared with overnight stay [13].

Contemporary patient-information and guideline sources similarly describe lumpectomy as a predominantly outpatient procedure with same-day discharge in most cases.

The strong influence of age, BMI, and overall surgical complexity in our model also aligns with prior work showing that higher BMI, older age, greater comorbidity burden, and longer operations predict extended LOS after breast and chest wall procedures [1].

These findings support the concept that personalized perioperative planning—incorporating enhanced recovery (ERAS) principles such as multimodal analgesia, early mobilization, and standardized discharge criteria—can meaningfully shorten LOS without compromising safety. ERAS pathways for breast reconstruction have repeatedly been associated with reduced opioid use and shorter hospital stays for both implant-based and autologous reconstruction [14].

Our feature-importance analysis further suggests that reconstruction technique and overall reconstructive “burden” (e.g., flap vs. implant, larger procedures) influence postoperative trajectories, consistent with studies showing that more complex microsurgical reconstructions carry longer baseline LOS and higher resource use than simpler implant-based approaches, even when optimized protocols are applied.

### 4.6. Integration into Daily Clinical Practice

The findings from this study support the practical integration of machine learning tools into routine surgical planning:

1. Preoperative counseling: ML-based LOS prediction could help set expectations, improving patient satisfaction and shared decision-making.
2. Resource allocation: Hospitals can anticipate postoperative bed occupancy more accurately.
3. Enhanced recovery protocols: Patients flagged as high risk for prolonged LOS may benefit from targeted interventions.
4. Cost optimization: Predictive risk stratification facilitates smarter distribution of nursing, physiotherapy, and social work resources.

### 4.7. The Role of Machine Learning in Predictive Modeling

Machine learning (ML), a methodological subset of artificial intelligence (AI), is increasingly used in healthcare to model complex clinical data. Modern AI systems excel at recognizing complex patterns across large datasets—patterns often invisible to traditional statistical methods or human perception. In surgical oncology, machine-learning applications include predicting complications, identifying high-risk patients, and supporting perioperative planning and assisting with intraoperative navigation. As our results demonstrate, machine-learning–based models bring added value when clinical data are heterogeneous, multidimensional, and nonlinear—conditions that accurately describe real-world breast cancer care.

### 4.8. Strengths and Limitations

Strengths of this study include the use of real-world, granular clinical and surgical data; inclusion of consecutive patients over a two-year interval; comparison of multiple ML algorithms with traditional regression; and transparent methodology aligned with STROBE standards. Limitations include the single-center design, which may affect generalizability; moderate sample size; missing values in certain pathology variables; and the retrospective nature of the data. Additionally, the study did not include external validation on an independent cohort, and classification models showed limited performance for predicting prolonged LOS [10,11,12]. The threshold defining prolonged LOS was institution-specific and may not generalize across healthcare systems; alternative cutoffs or percentile-based definitions may yield different classification performance.

This study relied exclusively on internal validation. No external validation cohort was available, and therefore, the reported performance metrics should not be interpreted as estimates of out-of-sample generalizability. While ensemble machine-learning methods were applied, extensive internal validation was used to reduce overfitting. These results should be interpreted as exploratory and hypothesis-generating, and external validation in larger, multicenter cohorts is required before clinical deployment. Simple median and mode imputation may influence model stability and feature-importance estimates by reducing variability and by not explicitly modeling informative missingness. While appropriate for this moderate-sized retrospective cohort, more advanced imputation strategies or missingness indicators may yield different results and should be considered in future studies.

## 5. Conclusions

This study demonstrates that machine-learning models—particularly Random Forest regression—substantially outperform classical linear regression in predicting postoperative LOS after breast cancer surgery. LOS is influenced primarily by age, surgical complexity, tumor burden, and reconstructive techniques, whereas classical tumor biology plays a smaller role. Given the clinical and economic relevance of LOS, these findings highlight the potential of machine learning to guide perioperative planning, optimize hospital resource utilization, and ultimately improve patient care. Future research should include multicenter cohorts and external validation to further refine predictive models and expand their applicability to diverse surgical practice settings.

## Figures and Tables

**Table 1:** Study group characteristics.

Characteristic	Value
Number of patients	198
Age, years—median (IQR)	53.5 (45.0–65.0)
BMI, kg/m^2^—median (IQR)	26.1 (22.8–29.8)
Smoking status—n (%)	Data heterogeneous; approx. ~10–15% recorded as smokers
Cardiovascular comorbidities—n (%)	97 (49.0%)
Diabetes mellitus—n (%)	47 (23.7%)
Tumor laterality—n (%)	Right: 54.0%; Left: 46.0%
ER-positive—n (% of known)	84.8%
PR-positive—n (% of known)	81.2%
HER2-positive—n (% of known)	20.2%
Ki-67, %—median (IQR)	20 (10–30)
High Ki-67 (≥20%)—n (% of known)	51.7%
pT stage—n (%)	pT1: ~35%; pT2: ~45%; pT3+: ~20% (approx. based on dataset distribution)
Nodal involvement (pN1–3)—n (%)	~35–40% (approx.)
Multicentric/multifocal—n (%)	~20–25%
Neoadjuvant chemotherapy—n (%)	85 (42.9%)
Breast-conserving surgery—n (%)	18 (9.1%)
Mastectomy without reconstruction—n (%)	60 (30.3%)
Mastectomy with immediate reconstruction—n (%)	40 (20.2%)
Other surgical combinations—n (%)	80 (40.4%)
Sentinel lymph node biopsy performed—n (%)	51 (25.8%)
Axillary lymphadenectomy—n (%)	90 (45.5%)
Length of stay, days—median (IQR)	7 (5–10)
LOS in breast-conserving surgery—median (IQR)	3 (2–3)
LOS in mastectomy without reconstruction—median (IQR)	7 (5–10)
LOS in mastectomy with immediate reconstruction—median (IQR)	8 (6.8–11.3)

**Table 2:** Performance of Regression Models for Predicting Length of Stay (LOS).

Model	MAE (Days)	RMSE (Days)	R^2^	Cross-Validated MAE (Days)
Linear Regression	8.63	10.73	–8.17	5.46
Random Forest Regressor	**2.31**	**2.82**	**0.37**	**3.04**
Gradient Boosting Regressor	2.63	3.15	0.21	3.07

Interpretation: The Random Forest regressor demonstrated the best overall performance, with the lowest MAE and RMSE and highest R^2^.

**Table 3:** Feature Importance from the Best Regression Model (Random Forest).

Rank	Variable	Importance
1	Age (Varsta_num)	Highest
2	Primary surgical procedure type	High
3	Implant reconstruction technique (submuscular/combined LD flap + implant)	High
4	BMI (BMI_num)	Moderate–high
5	Weight (Greutate_num)	Moderate–high
6	BRCA status	Moderate
7	Implant capacity (Capacitate)	Moderate
8	Ki-67 (%)	Moderate
9	Pathological tumor size (pT)	Moderate
10	ER/PR/HER2	Low

Interpretation: Age and surgical complexity were the strongest predictors of LOS, while receptor status contributed minimally.

**Table 4:** Classification Model Performance for Prolonged LOS (≥10 days).

Model	AUC	Accuracy	Sensitivity	Specificity	Cross-Validated AUC
Logistic Regression	0.545	0.65	0.00	0.93	0.535
Random Forest Classifier	0.589	0.675	0.00	0.96	0.660
Gradient Boosting Classifier	0.579	0.675	0.17	0.89	0.654

Interpretation: All classification models showed modest discrimination, with low sensitivity. Random Forest had the highest test AUC and cross-validated AUC, while Gradient Boosting showed slightly better sensitivity.

## Data Availability

The data is available on demand from the corresponding author.

## Abbreviations

The following abbreviations are used in this manuscript:

AI	Artificial Intelligence
AUC	Area Under the (Receiver Operating Characteristic) Curve
BCS	Breast-Conserving Surgery
BMI	Body Mass Index
BRCA	Breast Cancer Susceptibility Gene (BRCA1/BRCA2)
CNAS	Casa Națională de Asigurări de Sănătate (Romanian National Health Insurance House)
DCIS	Ductal Carcinoma in Situ
DRG	Ductal Carcinoma in Situ
ER	Estrogen Receptor
ERAS	Enhanced Recovery After Surgery
EHR	Electronic Health Record
HER2	Human Epidermal Growth Factor Receptor 2
HCUP	Healthcare Cost and Utilization Project
IHC	Immunohistochemistry
IQR	Interquartile Range
KI-67	Cellular Proliferation Index Ki-67
LD flap	Latissimus Dorsi Flap
LOS	Length of Stay
MAE	Mean Absolute Error
ML	Mean Absolute Error
NSQIP	National Surgical Quality Improvement Program
PR	Progesterone Receptor
pT	Pathological Tumor Size/Tumor Stage
RMSE	Root Mean Square Error
ROC	Receiver Operating Characteristic
SLNB	Sentinel Lymph Node Biopsy
STROBE	Strengthening the Reporting of Observational Studies in Epidemiology
